# Adipocyte fatty acid-binding protein 4 suppresses contraction of mouse ventricular myocytes via a calcium-independent pathway

**DOI:** 10.3389/fphys.2026.1682010

**Published:** 2026-03-10

**Authors:** Cuihua Wang, Daofeng You, Xuan Jiang, Shanshan Han, Fenghong Liu, Wei Wang, Mingqi Zheng

**Affiliations:** 1 Department of Cardiac Rehabilitation, The First Hospital of Hebei Medical University, Shijiazhuang, Hebei, China; 2 Emergency Department, The First Hospital of Hebei Medical University, Shijiazhuang, Hebei, China; 3 Arrhythmia Center, The First Hospital of Hebei Medical University, Shijiazhuang, Hebei, China; 4 Cardiovascular Disease Research Institute, Jiangxi Provincial People’s Hospital, Nanchang, Jiangxi, China

**Keywords:** ventricular myocytes, FABP4, calcium transients, contractility, myofilament sensitivity

## Abstract

**Objective:**

Adipocyte Fatty Acid-Binding Protein 4 (FABP4) exerts a direct negative inotropic effect on cardiac muscle, but the underlying cellular mechanisms remain elusive. This study aimed to dissect the specific effects of FABP4 on the contractility and calcium (Ca^2+^) homeostasis of isolated mouse ventricular myocytes and to characterize the functional role and critical residues of its N-terminal domain.

**Methods:**

Contractility and intracellular Ca^2+^ transients were simultaneously measured in isolated adult mouse ventricular myocytes using an IonOptix system following acute application of recombinant human FABP4 or its synthetic N-terminal peptide (FABP4_aa1-20_). L-type Ca^2+^ current was assessed via the whole-cell patch-clamp technique. Dose-response curves were analyzed using non-linear regression, and site-directed mutagenesis (E15K) was performed to evaluate the functional importance of a key amino acid residue.

**Results:**

FABP4 inhibited myocyte contraction in a biphasic, dose-dependent manner, with a high-affinity (EC_50_ = 0.010 pM) and a low-affinity (EC_50_ = 0.120 nM) component. This inhibition was largely independent of Ca^2+^ handling, as Ca^2+^ transient amplitude was only weakly attenuated at higher concentrations (EC_50_ = 0.412 nM), and L-type Ca^2+^ current was unaffected. In stark contrast, the FABP4_aa1-20_ peptide also inhibited contraction (EC_50_ = 0.110 nM) but did so via a Ca^2+^-dependent pathway, robustly suppressing Ca^2+^ transients. Mutation of glutamic acid at position 15 (E15K) significantly attenuated the peptide’s inhibitory activity.

**Conclusion:**

Full-length FABP4 suppresses cardiomyocyte contractility primarily through a Ca^2+^-independent pathway, likely by reducing myofilament Ca^2+^ sensitivity. Conversely, its isolated N-terminal domain operates via a distinct, Ca^2+^-dependent mechanism. These findings reveal a complex dual-pathway regulation of cardiac function by FABP4 and identify its N-terminal region as a potential therapeutic target for mitigating obesity-related cardiac dysfunction.

## Background

The global obesity epidemic is a primary driver of cardiovascular complications, leading to an increased incidence of hypertension, coronary heart disease, and heart failure (Oikonomou and Antoniades, 2019). Adipose tissue, no longer considered merely an energy depot, functions as a critical endocrine organ by secreting a variety of bioactive molecules known as adipokines (Luo et al., 2016; Zorena et al., 2020). Among these, Adipocyte Fatty Acid-Binding Protein (A-FABP), or FABP4, has emerged as a key player. FABP4 belongs to the family of intracellular lipid-binding proteins that facilitate the transport and metabolism of long-chain fatty acids (Furuhashi and Hotamisligil, 2008; Lee et al., 2014). While abundant in adipocytes and macrophages, from which it is secreted, FABP4 expression has also been detected in other cell types, including endothelial cells and potentially cardiomyocytes (Thumser and Moore, 2014; Elmasri et al., 2009; Iso et al., 2013; Goto et al., 2013). However, whether FABP4 is endogenously expressed within cardiomyocytes remains a subject of debate due to conflicting reports (Lamounier-Zepter et al., 2009).

Beyond its role in lipid metabolism, FABP4 is a crucial mediator of inflammatory signaling and is strongly implicated in the pathology of obesity-related metabolic diseases, including insulin resistance and atherosclerosis (Hui et al., 2010). Clinical studies have consistently demonstrated a strong positive correlation between elevated serum FABP4 concentrations and the severity of coronary artery disease and heart failure (Huang et al., 2013; Liu et al., 2013). Consequently, FABP4 is now regarded as a valuable prognostic biomarker for acute coronary syndromes (Reiser et al., 2015; Tu et al., 2017). Preclinical evidence further supports its pathogenic role; genetic deletion or pharmacological inhibition of FABP4 protects mice from diet-induced obesity and atherosclerosis, highlighting its potential as a therapeutic target (Furuhashi et al., 2007; Cao, 2014).

Heart-type fatty acid-binding protein (FABP3), a close homolog of FABP4, is highly expressed in the myocardium and serves as a sensitive biomarker for acute myocardial infarction (Ye et al., 2018; Furuhashi et al., 2014). Given their high structural and sequence homology, it is notable that both proteins have been shown to exert negative inotropic effects. Lamounier-Zepter et al. first reported that FABP4 secreted from human adipocytes directly suppresses rat cardiomyocyte contraction, an effect mimicked by FABP3 (Lamounier-Zepter et al., 2009). They also observed that a peptide comprising the N-terminal 20 amino acids of FABP4 (FABP4_aa1-20_) retained significant cardio-suppressive activity. This study builds upon these foundational findings to dissect the precise cellular mechanisms by which FABP4 modulates ventricular myocyte function. We aimed to characterize the dose-dependent effects of FABP4 on both cardiomyocyte contractility and Ca^2+^ homeostasis and to investigate the functional role of its N-terminal domain.

## Methods

### Animal experiments

Male C57BL/6 mice (12–14 weeks old) were obtained from the Hebei Experimental Animal Centre. All animals were housed in a specific-pathogen-free (SPF) facility with a 12-h light/dark cycle, maintained at 23 °C–25 °C, and provided with *ad libitum* access to standard chow and water. All experimental procedures were approved by the Experimental Animal Ethics Committee of Hebei Medical University and were conducted in accordance with the ARRIVE guidelines (Percie du Sert et al., 2020).

### Mouse ventricular myocyte isolation

Adult mouse ventricular myocytes were isolated using a standard Langendorff perfusion protocol as previously described (Wang et al., 2015). Briefly, mice were anesthetized, and hearts were rapidly excised and cannulated via the aorta. Hearts were perfused with a Ca^2+^-free Tyrode’s solution (in mM: 137 NaCl, 5.4 KCl, 1 MgCl_2_, 10 glucose, 10 HEPES; pH 7.4 with NaOH) for 3 min, followed by enzymatic digestion with the same solution containing 0.4 g/L Type II collagenase (Roche, United States) for 20–25 min at 37 °C. The left ventricle was then minced and gently triturated to release single myocytes. Cells were filtered through a 200 µm mesh and resuspended in KB solution (in mM: 70 potassium glutamate, 20 KCl, 1 K_2_HPO_4_, 5 MgCl_2_, 20 taurine, 5 creatine, 5 HEPES, 20 glucose, 0.5 EGTA; pH 7.2 with KOH) for 40 min before gradual reintroduction of Ca^2+^.

### Immunoblot analyses

Total protein was extracted from myocardial tissue and isolated cardiomyocytes using RIPA buffer (Solarbio, R0010, China) supplemented with protease inhibitors. Protein concentrations were determined, and 20 µg of protein per lane was separated by 10% SDS-PAGE. Proteins were transferred to a PVDF membrane, which was then blocked for 2 h with 5% non-fat dry milk in TTBS. Membranes were incubated overnight at 4 °C with primary antibodies against FABP4 (1:1000, Santa Cruz), FABP3 (1:1000, Abcam), and β-actin (1:1000, Santa Cruz). After washing, membranes were incubated with appropriate HRP-conjugated secondary antibodies (1:1000) for 1 h. Protein bands were visualized using an enhanced chemiluminescence (ECL) system (Vilber Lourmat, France) and quantified with ImageJ software.

### Quantitative RT-PCR

Total RNA was extracted using an OMEGA extractor kit (Omega Bio-tek, R6834, United States), and cDNA was synthesized with an M-MLV first-strand synthesis kit (Invitrogen, cat. no. 18080051). Real-time PCR was performed using SYBR Green qPCR Master Mix (Thermo, cat. no. A25742) on an ABI 7500 Fast system. Primers are listed in Table 1. Relative gene expression was calculated using the 2^−ΔΔCt^ method with 18S rRNA as the housekeeping gene.

**TABLE 1:** Primer sequences used for quantitative RT-PCR.

Gene	Forward primer sequence (5′–3′)	Reverse primer sequence (5′–3′)
FABP3	AGTCACTGGTGACGCTGGACG	AGGCAGCATGGTGCTGAGCTG
FABP4	TTGGTCACCATCCGGTCAGA	CCTGTCGTCTGCGGTGATTT
18SrRNA	CGCCGCTAGAGGTGAAATTC	CCAGTCGGCATCGTTT ATGG

The thermal cycling program was as follows: denaturing at 95 °C for 10 s, followed by 40 cycles of 95 °C for 5 s, annealing at 65 °C for 30 s, and extension at 72 °C for 20 s.

### Myocyte contractility and calcium transient measurements

Myocytes were loaded with 1 µM Fluo-4 a.m. (Invitrogen, F14201) and 0.02% Pluronic F-127 (Invitrogen, P3000MP) for 40 min for Ca^2+^ imaging. Cells were placed in a perfusion chamber on the stage of an inverted microscope coupled to an IonOptix myocyte contractility and fluorescence system (IonOptix, United States). Myocytes were field-stimulated at 1 Hz. Cell shortening (sarcomere length) and Ca^2+^ transients (Fluo-4 fluorescence) were recorded simultaneously. For dose-response experiments, baseline data were recorded in normal Tyrode’s solution (1.8 mM Ca^2+^), followed by 10-min incubations with increasing concentrations of recombinant human FABP4 (Cayman Chemical, cat. no. 10005067), FABP4_aa1-20_ peptides, or mutated peptides (Sangon Biotech, China). Only rod-shaped myocytes with clear striations and stable baseline contractions were selected for analysis.

### Electrophysiological measurements

L-type Ca^2+^ current (I_Ca,L_) was recorded using the whole-cell patch-clamp technique with an Axon 200B amplifier (Axon Instruments). The pipette solution contained (in mM): 110 CsCl, 20 TEA-Cl, 5 Mg-ATP, 10 EGTA, 10 Glucose, 5 HEPES (pH 7.2 with CsOH). The external solution was normal Tyrode’s. I_Ca,L_ was elicited by 500 m depolarizing steps from a holding potential of −40 mV to +50 mV in 10 mV increments.

### Statistical analysis

Data are presented as mean ± standard error of the mean (SEM). Statistical comparisons between two groups were performed using an independent-sample t-test or paired t-test as appropriate. For multiple group comparisons, one-way ANOVA followed by a *post* *hoc* test was used. A p-value of less than 0.05 was considered statistically significant. Dose-response curves were fitted using non-linear regression (log(agonist) vs. response) in GraphPad Prism 9.0. A two-site binding model was compared to a one-site model using an F-test to determine the best fit for the data. All analyses were performed with IBM SPSS Statistics (Version 21.0) and GraphPad Prism. In all figure legends, “N” denotes the number of animals (biological replicates), and “n” denotes the number of cells (technical replicates).

## Results

### FABP4 is expressed in mouse myocardial tissue and ventricular myocytes

**FIGURE 1:**
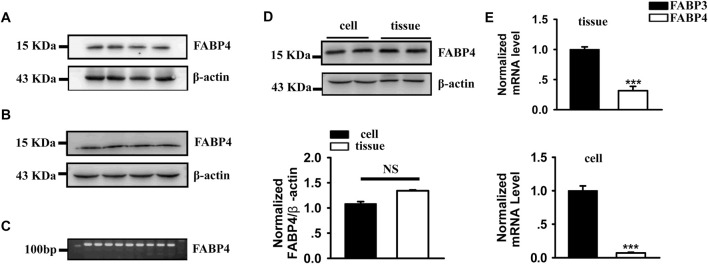
FABP4 expression in mouse myocardial tissue and cardiomyocytes. **(A,B)** Representative immunoblots demonstrating FABP4 protein expression in whole myocardial tissue and isolated ventricular myocytes. **(C)** Agarose gel electrophoresis of qRT-PCR products for FABP4. **(D)** Relative mRNA expression of FABP4 shows no significant difference between whole myocardial tissue (Tissue) and isolated ventricular myocytes (Cell). **(E)** Comparison of FABP3 and FABP4 mRNA expression reveals significantly higher levels of FABP3 in both tissue and cells. Data are mean ± SEM. (N = 4 animals, ***P < 0.001). Statistical significance was determined using a Student’s t-test.

To confirm the relevance of FABP4 in our model, we first assessed its expression. Immunoblot analysis detected FABP4 protein in whole myocardial tissue lysates and, importantly, in purified adult ventricular myocytes (Figures 1A,B). Similarly, qRT-PCR analysis showed comparable levels of FABP4 mRNA in both whole tissue and isolated myocytes (Figures 1C,D). As expected, the expression of FABP3, the predominant cardiac isoform, was significantly higher than FABP4 in both myocardial tissue and cardiomyocytes (Figure 1E). These data confirm that cardiomyocytes are a potential direct target for both endogenous and exogenous FABP4.

### FABP4 induces a biphasic, dose-dependent inhibition of myocyte contraction

**FIGURE 2:**
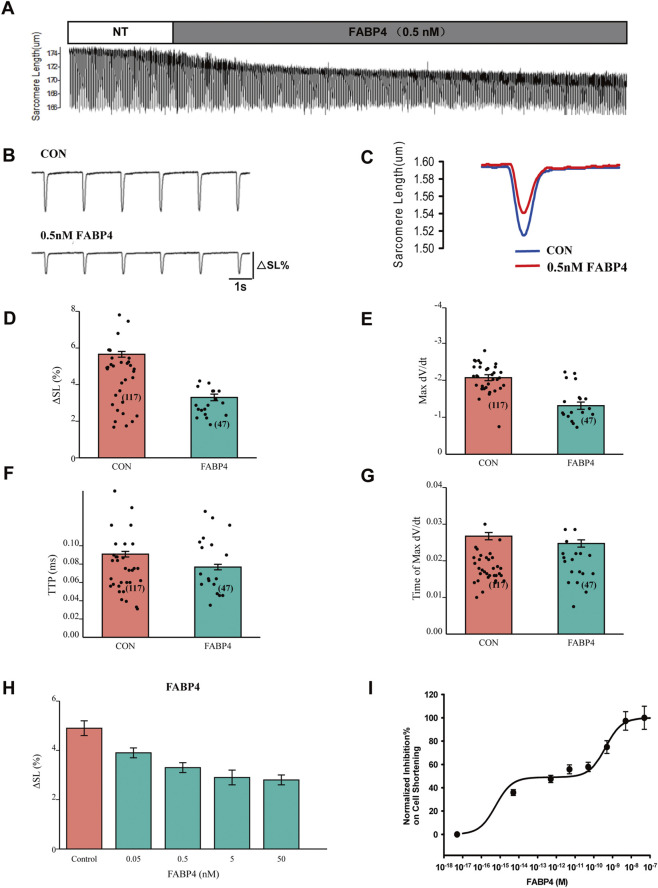
Dose-dependent inhibition of cardiomyocyte contraction by FABP4. **(A–C)** Representative traces of cardiomyocyte contraction before (NT, Normal Tyrode) and after perfusion with 0.5 nM FABP4. **(D)** Contraction amplitude (ΔSL%) is significantly inhibited by 0.5 nM FABP4. **(E)** Maximal rate of contraction (Max -dL/dt) is reduced. **(F)** Time to peak contraction (TTP) is shortened. **(G)** Time required for maximal contraction rate is unchanged. For D-G, CON: n = 117 cells from N = 8 animals; FABP4: n = 47 cells from N = 5 animals. Statistical significance was determined using an unpaired Student’s t-test (*P < 0.05, ***P < 0.001). **(H)** Bar chart showing the effect of increasing FABP4 concentrations on contractile amplitude. For H, the bars represent control and incubations with 0.05 nM, 0.5 nM, 5 nM, and 50 nM FABP4, respectively. **(I)** Dose-response curve of FABP4-induced inhibition of contractile amplitude. Curve fitting with a two-site model yielded a high-affinity EC_50_ of 0.010 pM and a low-affinity EC_50_ of 0.120 nM. Data points represent mean ± SEM from at least N = 4 animals per concentration.

We next investigated the direct effect of exogenous FABP4 on cardiomyocyte contractility. Acute application of FABP4 (0.5 nM) significantly reduced the contraction amplitude (% shortening) compared to control conditions (Figures 2A–D). This reduction in contractility was primarily driven by a decrease in the maximal rate of contraction (Max -dL/dt) and a shortened time to peak contraction (TTP) (Figures 2E,F), while the time to reach the maximal contraction rate was unaffected (Figure 2G). To characterize this effect further, we performed a comprehensive dose-response analysis over a wide concentration range (10^−8^ nM–100 nM). As shown in Figure 2H, FABP4 inhibited myocyte contraction in a dose-dependent manner, with the response reaching a plateau at concentrations above 50 nM. Interestingly, fitting the dose-response data to a two-site binding model, which provided a statistically superior fit over a one-site model (F-test, p < 0.05), revealed a potential biphasic inhibitory mechanism (Figure 2I). This model yielded a high-affinity component with an estimated EC_50_ of 0.010 pM and a low-affinity component with an EC_50_ of 0.120 nM.

### Inhibition of contraction by FABP4 is largely independent of calcium transients

**FIGURE 3:**
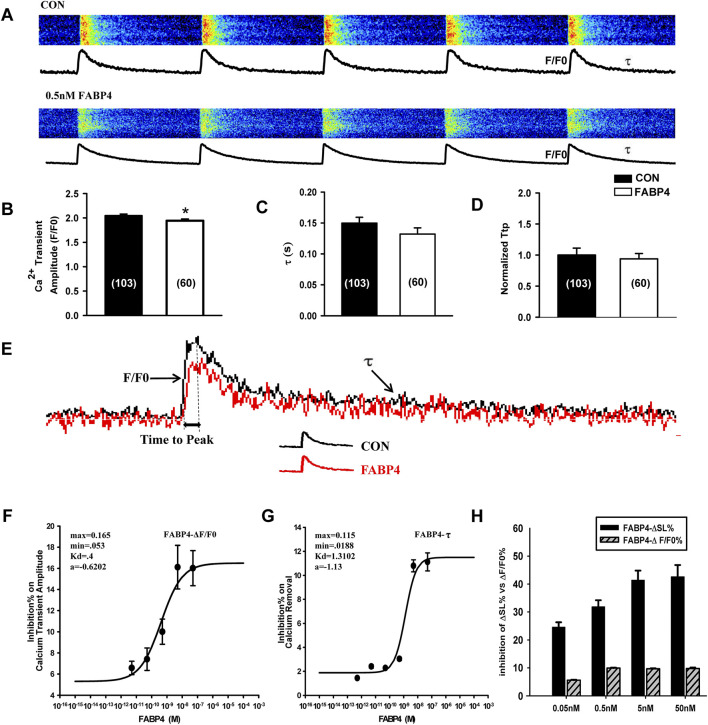
Effect of FABP4 on calcium transients in mouse ventricular myocytes. **(A)** Representative line-scan images of Ca^2+^ transients. **(B)** Ca^2+^ transient amplitude (F/F_0_) is slightly but significantly reduced by 0.5 nM FABP4. **(C)** The rate of Ca^2+^ recovery (τ) and **(D)** normalized time to peak (TTP) are not significantly altered. **(E)** Superimposed average Ca^2+^ transient traces for control (black) and FABP4-treated (red) cells. For B-E, CON: n = 103 cells from N = 7 animals; FABP4: n = 60 cells from N = 5 animals. Statistical significance was determined using an unpaired Student’s t-test (*P < 0.05). **(F,G)** Dose-response curves for FABP4 inhibition of Ca^2+^ transient amplitude (EC_50_ = 0.412 nM) and Ca^2+^ removal rate (EC_50_ = 1.312 nM). **(H)** Comparison of the dose-dependent inhibition of contractile amplitude (ΔSL%) versus Ca^2+^ transient amplitude (ΔF/F_0_), highlighting the mechanistic dissociation. Data are mean ± SEM from N = 4-5 animals per concentration.

To determine if the negative inotropic effect of FABP4 was mediated by alterations in Ca^2+^ handling, we simultaneously measured Ca^2+^ transients. At a concentration of 0.5 nM, which robustly inhibits contraction, FABP4 induced only a minor but statistically significant decrease in the Ca^2+^ transient amplitude (F/F_0_) (Figures 3A,B). The rate of Ca^2+^ decay (τ), an index of SERCA2a activity, and the time to peak Ca^2+^ were not significantly altered at this concentration (Figures 3C–E). Dose-response analysis revealed that FABP4 did inhibit Ca^2+^ transient amplitude and decay rate in a concentration-dependent manner, but with much lower potency (EC_50_ of 0.412 nM for amplitude and 1.312 nM for decay) compared to its effect on contractility (Figures 3F,G). A direct comparison highlights this mechanistic disparity: substantial inhibition of contraction occurs at concentrations where the effect on Ca^2+^ transients is minimal (Figure 3H). This suggests that the primary mechanism for FABP4-induced contractile suppression is independent of changes in global intracellular Ca^2+^ dynamics.

### FABP4 does not modulate L-type Ca^2+^ channel activity

**FIGURE 4:**
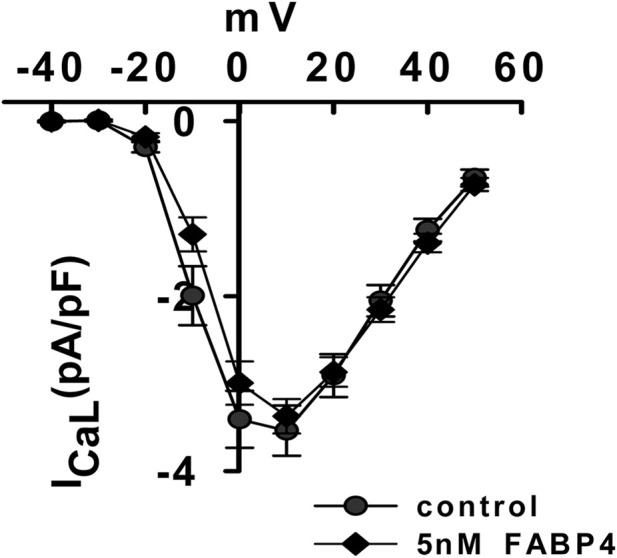
FABP4 does not affect L-type Ca^2+^ current. Current-voltage (I–V) relationship for L-type Ca^2+^ current (I_Ca,L_) in cardiomyocytes before (Control) and after exposure to 5 nM FABP4. No significant difference was observed. Data are mean ± SEM (Control: n = 15 cells from N = 4 animals; FABP4: n = 26 cells from N = 5 animals). Comparisons at each voltage step were made using a Student’s t-test.

To exclude triggered activity from Ca^2+^ influx via L-type Ca^2+^ channels as a potential mechanism, we measured I_Ca,L_ using the patch-clamp technique. Application of 5 nM FABP4, a concentration that elicits a strong low-affinity contractile response, had no significant effect on the current-voltage (I-V) relationship of I_Ca,L_ (Figure 4). This result further supports the conclusion that FABP4’s negative inotropic effect is not mediated by modulation of the principal Ca^2+^ entry pathway.

### The N-terminal peptide FABP4_aa1-20_ inhibits contraction via a calcium-dependent mechanism

**FIGURE 5:**
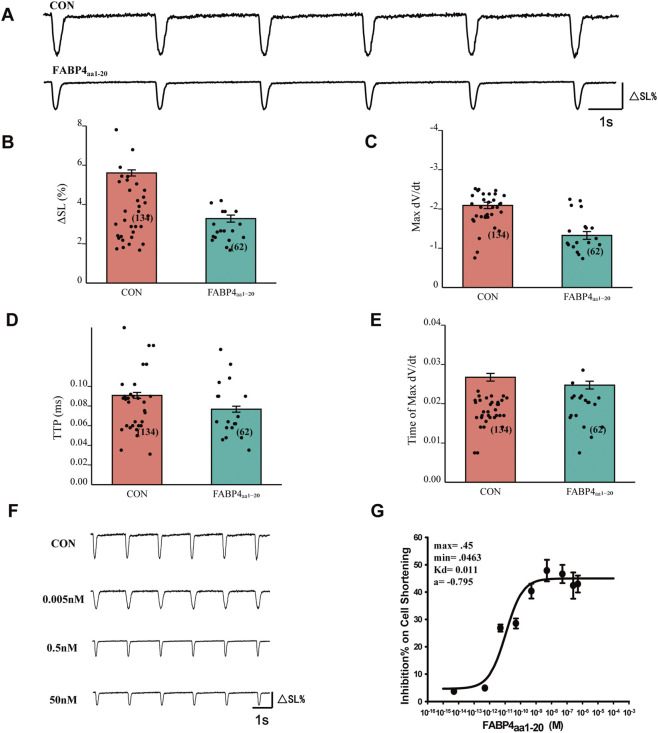
The N-terminal peptide FABP4_aa1-20_ inhibits cardiomyocyte contractile function. **(A)** Representative traces of myocyte contraction showing inhibition by 0.5 nM FABP4_aa1-20_. **(B–E)** FABP4_aa1-20_ significantly reduces contractile amplitude (ΔSL%), maximal rate of contraction (Max -dL/dt), and time to peak (TTP). CON: n = 134 cells from N = 8 animals; FABP4_aa1-20_: n = 62 cells from N = 5 animals. Statistical significance was determined using an unpaired Student’s t-test (***P < 0.001). **(F,G)** Dose-response curve for FABP4_aa1-20_ inhibition of contraction, yielding a monophasic fit with an EC_50_ of 0.110 nM. Data are mean ± SEM from N = 4-5 animals per concentration.

Previous work suggested that the N-terminal region of FABP4 is functionally important. We tested this by applying a synthetic peptide corresponding to the first 20 amino acids of FABP4 (FABP4_aa1-20_). Similar to the full-length protein, FABP4_aa1-20_ (0.5 nM) significantly inhibited myocyte contraction amplitude, maximal contraction rate, and time to peak (Figures 5A–E). Dose-response curve fitting showed that FABP4_aa1-20_ inhibited contraction with a monophasic, low-affinity mechanism, yielding an EC_50_ of 0.110 nM, nearly identical to the low-affinity component of the full-length protein (Figures 5F,G).

**FIGURE 6:**
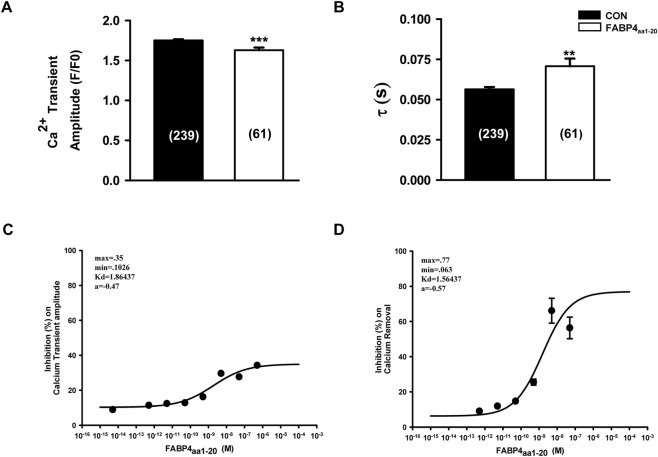
FABP4_aa1-20_ inhibits calcium transients in a dose-dependent manner. **(A,B)** Application of 0.5 nM FABP4_aa1-20_ significantly reduces Ca^2+^ transient amplitude (F/F_0_) and slows the Ca^2+^ recovery rate (τ). CON: n = 239 cells from N = 10 animals; FABP4_aa1-20_: n = 61 cells from N = 5 animals. Statistical significance was determined using an unpaired Student’s t-test (**P < 0.01, ***P < 0.001). **(C,D)** Dose-response curves for FABP4_aa1-20_ inhibition of Ca^2+^ transient amplitude (EC_50_ = 1.860 nM) and Ca^2+^ removal rate (EC_50_ = 1.564 nM). Data are mean ± SEM from N = 4-5 animals per concentration.

In stark contrast to the full-length protein, however, the inhibitory effect of FABP4_aa1-20_ was strongly associated with changes in Ca^2+^ handling. The peptide robustly inhibited both the Ca^2+^ transient amplitude (EC_50_ = 1.860 nM) and the rate of Ca^2+^ decay (EC_50_ = 1.564 nM) in a dose-dependent manner (Figures 6A–D). The similar potency for inhibiting both contraction and Ca^2+^ transients suggests that, unlike the full-length protein, the isolated N-terminal peptide suppresses contractility primarily through a Ca^2+^-dependent mechanism.

### Glutamate at position 15 is a key residue for the inhibitory activity of FABP4_aa1-20_

**FIGURE 7:**
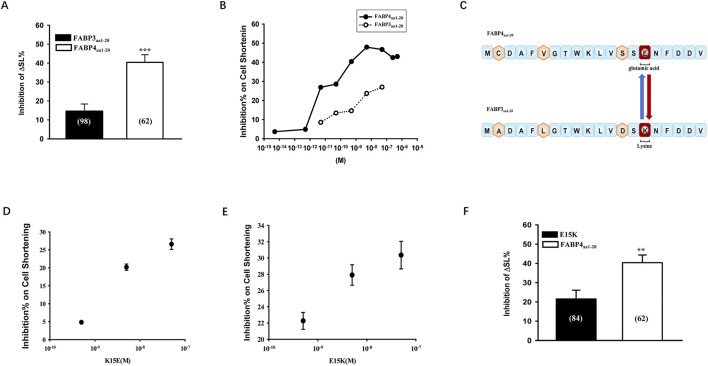
Glutamate at position 15 is a key determinant of FABP4_aa1-20_ inhibitory activity. **(A)** Comparison of contractile inhibition by 0.5 nM FABP3_aa1-20_ and FABP4_aa1-20_. Statistical significance was determined using a Student’s t-test (***P < 0.001). **(B)** Dose-dependent comparison showing greater potency of FABP4_aa1-20_. **(C)** Amino acid sequences of the N-terminal peptides, highlighting the key difference at position 15 (K vs. **(E)** and the engineered mutants (K15E and E15K). **(D,E)** Concentration-dependent effects of a gain-of-function mutant (K15E) and a loss-of-function mutant (E15K). **(F)** The E15K mutation significantly attenuates the inhibitory effect of FABP4_aa1-20_ on contraction compared to the wild-type peptide at 0.5 nM. Statistical significance was determined using a Student’s t-test (**P < 0.01). Data are mean ± SEM from N = 5-6 animals for each group.

The amino acid sequences of FABP3 and FABP4 are highly homologous, with only four differences in the first 20 residues. One key difference is at position 15, which is a lysine (K, basic) in FABP3 and a glutamic acid (E, acidic) in FABP4. We confirmed that FABP4_aa1-20_ was a significantly more potent inhibitor of contraction than FABP3_aa1-20_ (Figures 7A,B). To investigate the importance of residue 15, we performed site-directed mutagenesis. Swapping the glutamic acid in FABP4_aa1-20_ for a lysine (E15K) significantly attenuated its inhibitory effect by approximately 50% (Figures 7C,F). Conversely, substituting the lysine in FABP3_aa1-20_ with a glutamic acid (K15E) enhanced its inhibitory activity (Figures 7D,E). These results strongly suggest that the negatively charged glutamate at position 15 is a critical determinant of the cardio-inhibitory function of the FABP4 N-terminal domain.

## Discussion

This study provides novel insights into the cellular mechanisms underlying the direct negative inotropic effects of FABP4 on ventricular myocytes. Our principal findings are threefold: 1) Full-length FABP4 inhibits cardiomyocyte contraction through a primary mechanism that is independent of changes in L-type Ca^2+^ current or global Ca^2+^ transients, suggesting a direct effect on sarcomeric proteins. 2) The dose-response relationship of FABP4 appears to be biphasic, though the physiological relevance of the ultra-high-affinity component requires further investigation. 3) In contrast, the N-terminal peptide FABP4_aa1-20_ recapitulates the low-affinity inhibitory effect but does so via a Ca^2+^-dependent pathway, with residue Glu15 being critical for its activity.

Our confirmation of FABP4 expression in isolated cardiomyocytes aligns with some previous reports (Zhang et al., 2016; Lee et al., 2020) but contrasts with others that localized it primarily to cardiac endothelial cells (Lamounier-Zepter et al., 2009; Zhou et al., 2015). These discrepancies may arise from methodological differences, but our data establish a basis for autocrine or intracrine actions of FABP4 within the cardiomyocyte itself, in addition to its known endocrine effects.

A key finding of our study is the dose-dependent inhibition of myocyte contraction by FABP4. The biphasic nature of this inhibition, as suggested by our statistical modeling, is provocative. It is important to contextualize our findings with reported FABP4 concentrations. In severe pathophysiological states, such as end-stage renal disease, circulating FABP4 levels can reach very high concentrations (∼300–400 ng/mL, or ∼20–27 nM) (Furuhashi et al., 2011). At these levels, the low-affinity inhibitory component we identified (EC_50_ ∼0.12 nM) would be fully saturated. This suggests that in advanced disease states with markedly elevated FABP4, this cardio-inhibitory mechanism would be maximally and persistently activated, contributing significantly to cardiac dysfunction rather than providing a modulated response. However, it is crucial to distinguish between systemic circulating concentrations and local concentrations within the cardiac microenvironment, as the latter are often significantly lower and mediate direct paracrine or autocrine effects (Furuhashi et al., 2014). Therefore, the low-affinity mechanism may be more relevant in conditions with mild to moderate elevations of FABP4, where it could be progressively engaged. In contrast, the physiological significance of the modeled high-affinity site (EC_50_ ∼10 fM) is intriguing. This ultra-high sensitivity, which is several orders of magnitude below typical circulating concentrations, may reflect a highly sensitive physiological signaling mechanism, potentially responding to low levels of FABP4 secreted locally by cardiac adipocytes or even the cardiomyocytes themselves (Zhang et al., 2016). Validation of this biphasic effect and its relation to local FABP4 concentrations will require further investigation.

**FIGURE 8:**
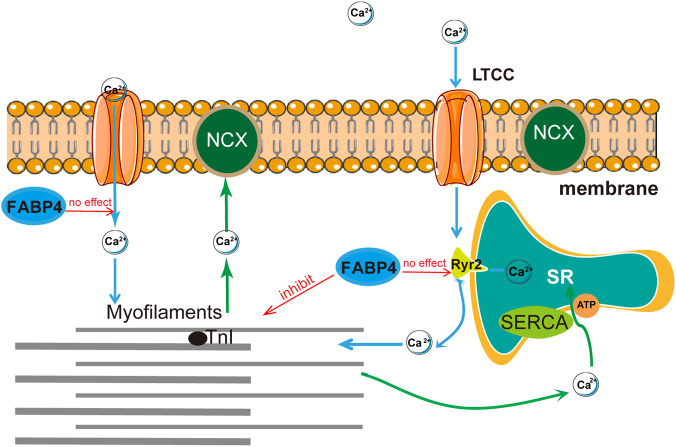
Proposed mechanism of FABP4-induced inhibition of cardiomyocyte contraction. Full-length FABP4 primarily suppresses contraction via a Ca^2+^-independent pathway, likely by decreasing the Ca^2+^ sensitivity of the myofilaments. This effect is independent of changes in L-type Ca^2+^ channels (I_Ca,L_) or sarcoplasmic reticulum (SR) Ca^2+^ release and reuptake. In contrast, the isolated N-terminal peptide (FABP4_aa1-20_) inhibits contraction via a Ca^2+^-dependent mechanism, suppressing SR Ca^2+^ release.

The most significant mechanistic insight is the dissociation between the contractile inhibition and Ca^2+^ handling by full-length FABP4. By excluding significant effects on I_Ca,L_ and demonstrating that robust contractile depression occurs at concentrations that minimally affect Ca^2+^ transients, our data strongly point towards the myofilaments as the primary target. FABP4 could directly or indirectly modulate the Ca^2+^ sensitivity of the sarcomere. This mechanism is employed by various signaling molecules that alter the phosphorylation status of myofilament proteins like troponin I (TnI) and myosin binding protein-C (Gorski et al., 2015; Ponnam et al., 2019; Harris, 2021). Future studies should therefore investigate whether FABP4 interacts with sarcomeric proteins or alters their post-translational modifications to decrease the force generated for a given amount of Ca^2+^. A schematic summary of our proposed mechanism is shown in Figure 8.

A particularly intriguing finding is the mechanistic divergence between full-length FABP4 and its N-terminal peptide. While FABP4_aa1-20_ mimicked the low-affinity inhibitory potency, its action was clearly Ca^2+^-dependent. This discrepancy suggests that the context of the full protein is critical. One possibility is that the C-terminal portion of FABP4 sterically hinders the N-terminus from accessing and modulating Ca^2+^-handling machinery (e.g., SERCA2a or RyR2). When liberated as a free peptide, the N-terminus may interact with these targets, leading to the observed suppression of Ca^2+^ transients. This highlights a fascinating structural-functional relationship that warrants further exploration.

Our mutagenesis data identified Glu15 as a key residue for the bioactivity of the FABP4 N-terminal domain. The substitution of this acidic residue with the basic lysine found in FABP3 dramatically reduced its inhibitory effect, suggesting that electrostatic interactions are crucial for its function. This finding provides a specific molecular target for the design of potential therapeutic inhibitors aimed at blocking the adverse cardiac effects of FABP4.

This study has several limitations. First, we used recombinant human FABP4 on mouse myocytes; although highly homologous, potential species-specific differences cannot be entirely excluded. Second, the L-type Ca2+ current was assessed at only one concentration of FABP4, and while this concentration was relevant to the low-affinity effect, we cannot rule out a role for ICa,L in the putative high-affinity mechanism. Furthermore, the most significant limitation is that while we confirmed the presence of FABP4 protein, we did not perform absolute quantification of its endogenous concentration in cardiomyocytes or the local cardiac microenvironment. As highlighted by the clear discrepancy between the effective concentrations used in our functional assays and the high systemic levels of FABP4 reported in pathological conditions, this absence of direct concentration data means that our interpretation of the physiological and pathological relevance of the observed effects remains, to some extent, speculative. Finally, our conclusions are based on *in vitro* experiments with isolated myocytes. *In vivo* studies are necessary to confirm these findings and understand their implications in the integrated physiological context of the heart.

In conclusion, this study demonstrates that FABP4 potently suppresses ventricular myocyte contraction primarily via a Ca^2+^-independent pathway, likely involving the modulation of myofilament sensitivity. Its N-terminal domain, however, acts through a distinct Ca^2+^-dependent mechanism. These findings unravel a new layer of complexity in the link between metabolism and cardiac function and identify the N-terminus of FABP4 as a potential therapeutic target to mitigate obesity-associated heart disease.

## Data Availability

The original contributions presented in the study are included in the article/Supplementary Material, further inquiries can be directed to the corresponding authors.
