# Supplementary figures and images for: Adipocyte fatty acid-binding protein 4 suppresses contraction of mouse ventricular myocytes via a calcium-independent pathway
Source: Front Physiol. 2026 Mar 10;17:1682010. doi: 10.3389/fphys.2026.1682010 (PMC13008700; doi:10.3389/fphys.2026.1682010)

Fig 1A-FABP4


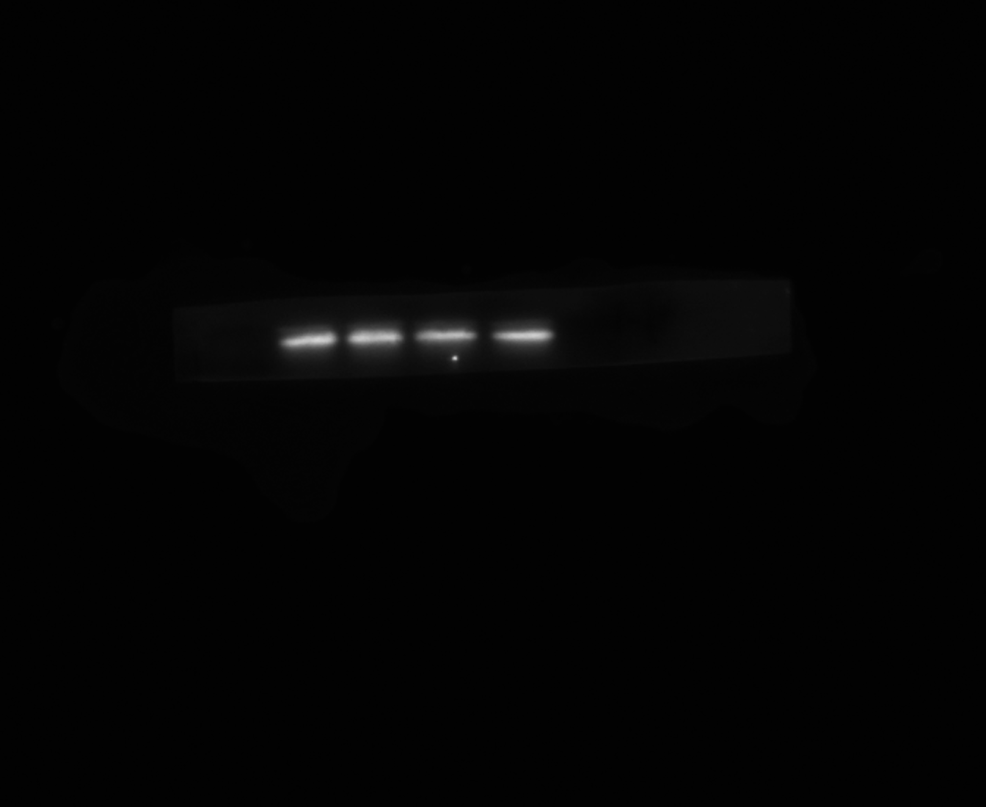


Fig 1A-β-actin


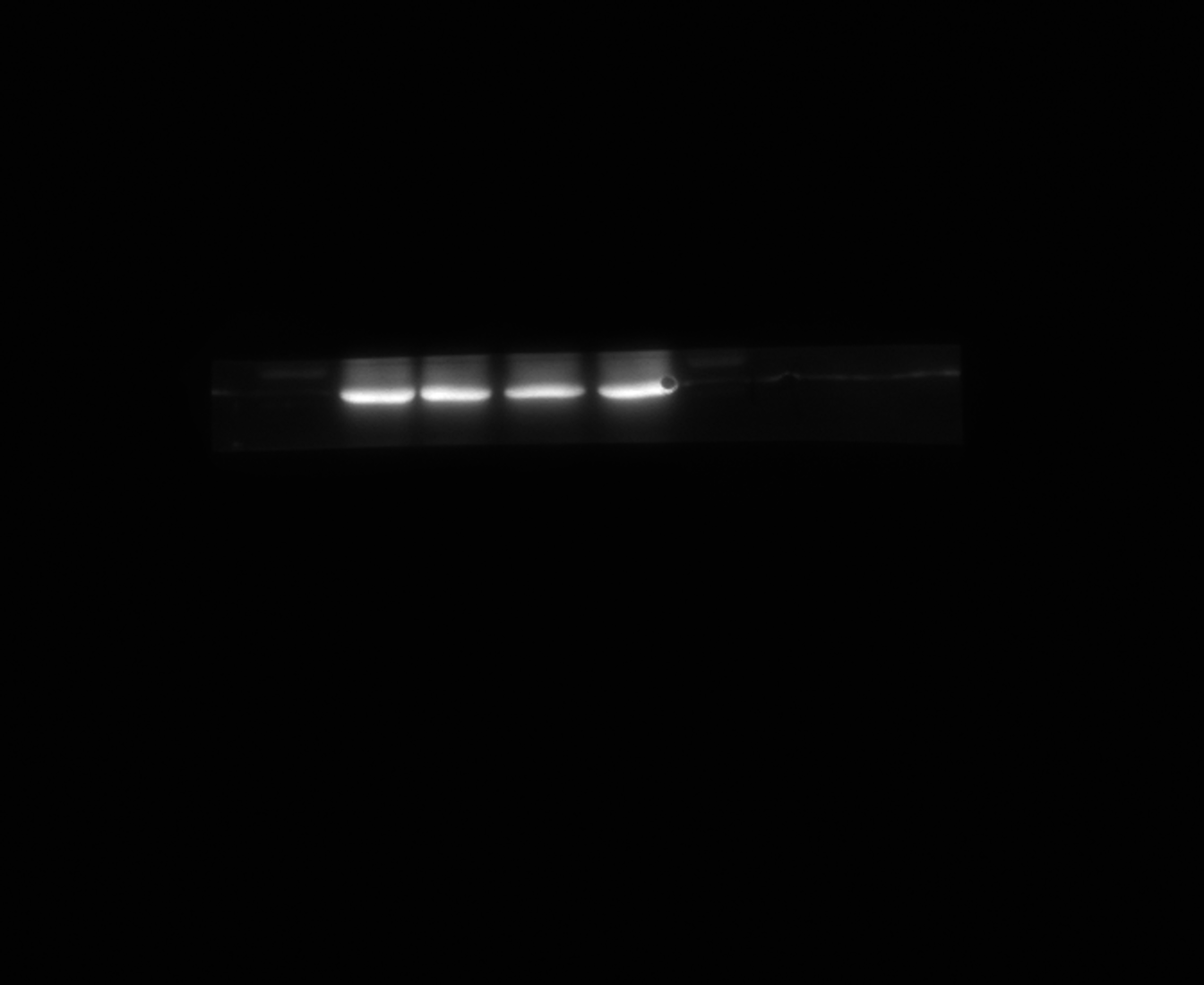


Fig 1B-FABP4


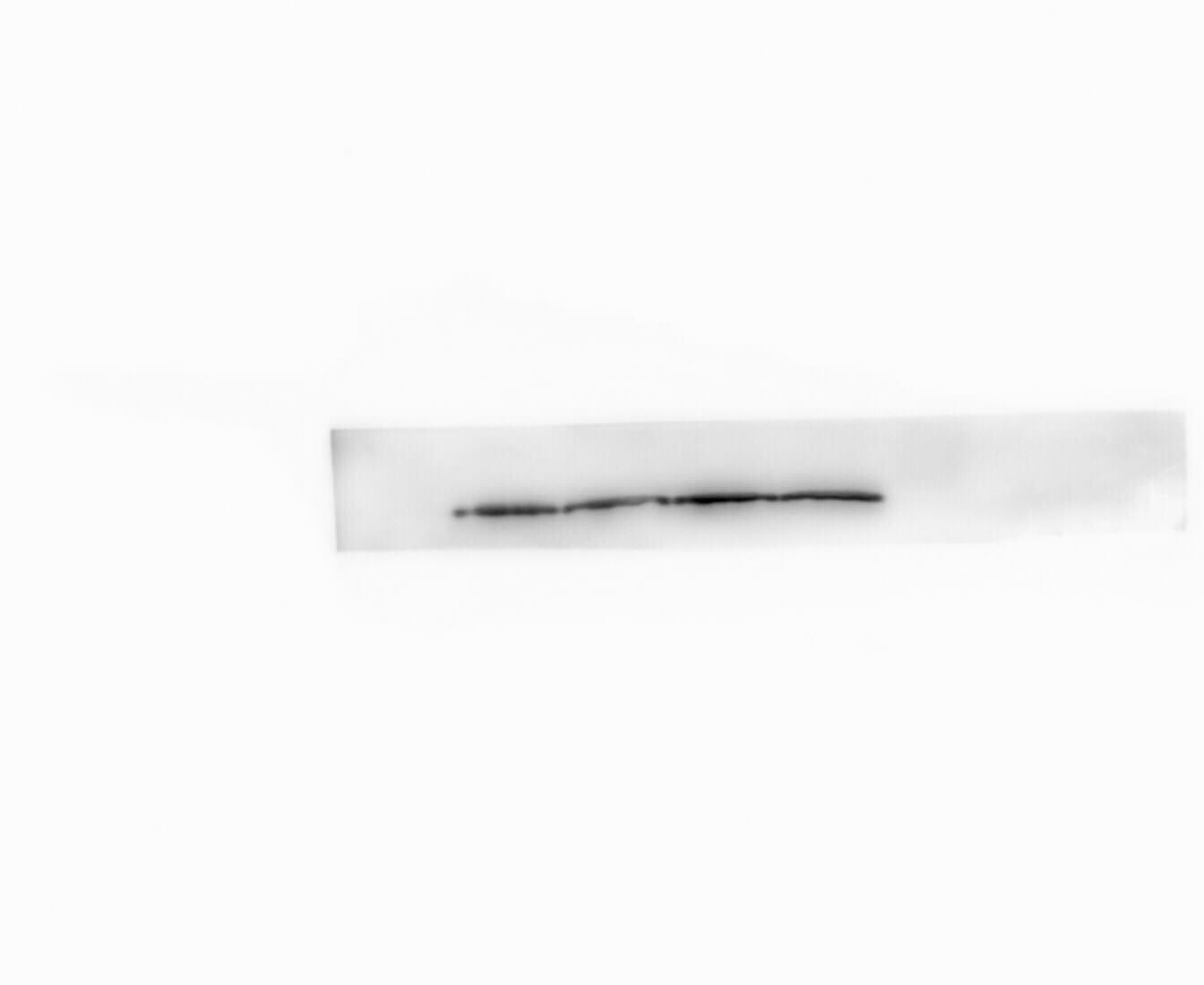


Fig 1B-β-actin


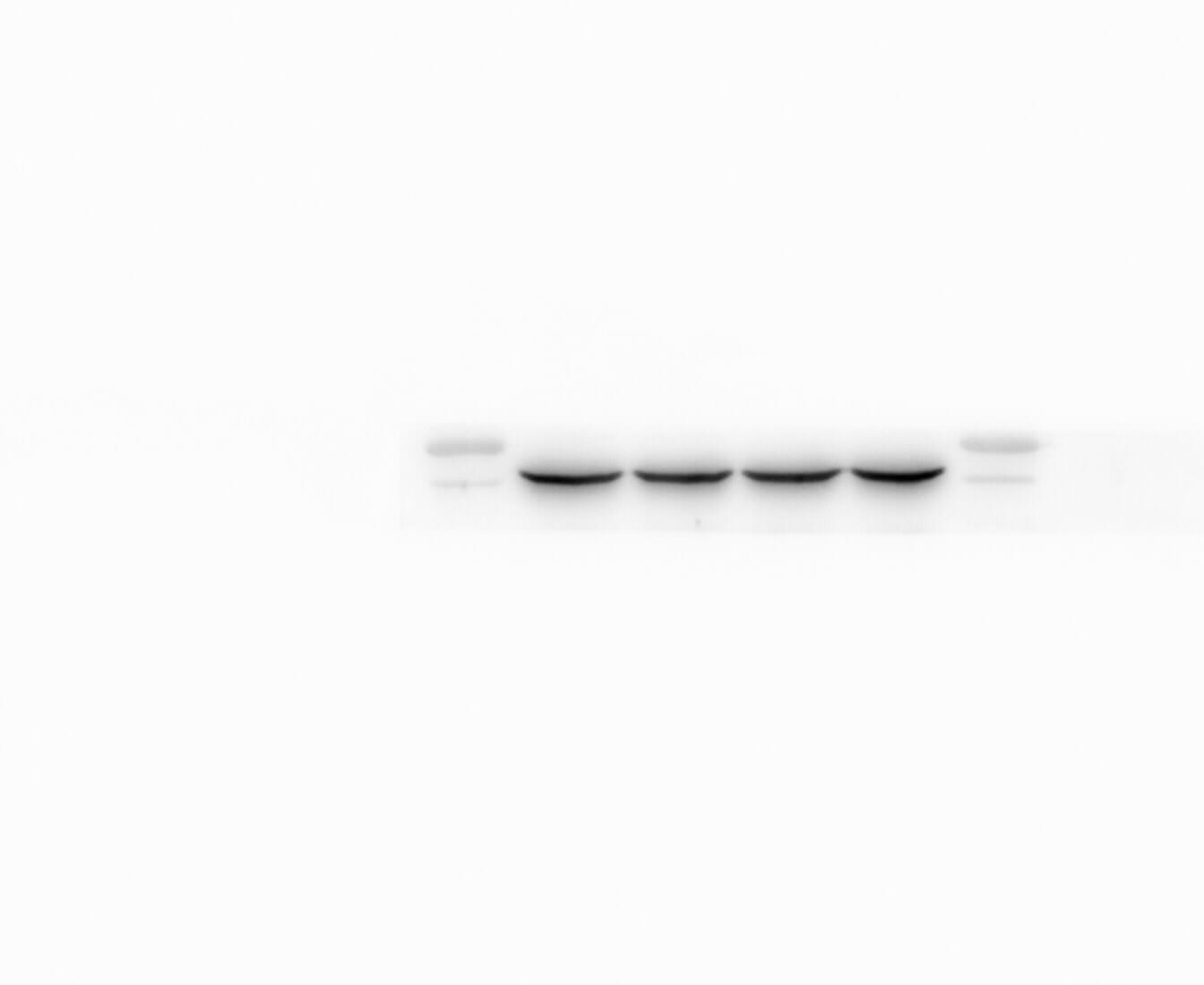


Fig 1D-FABP4


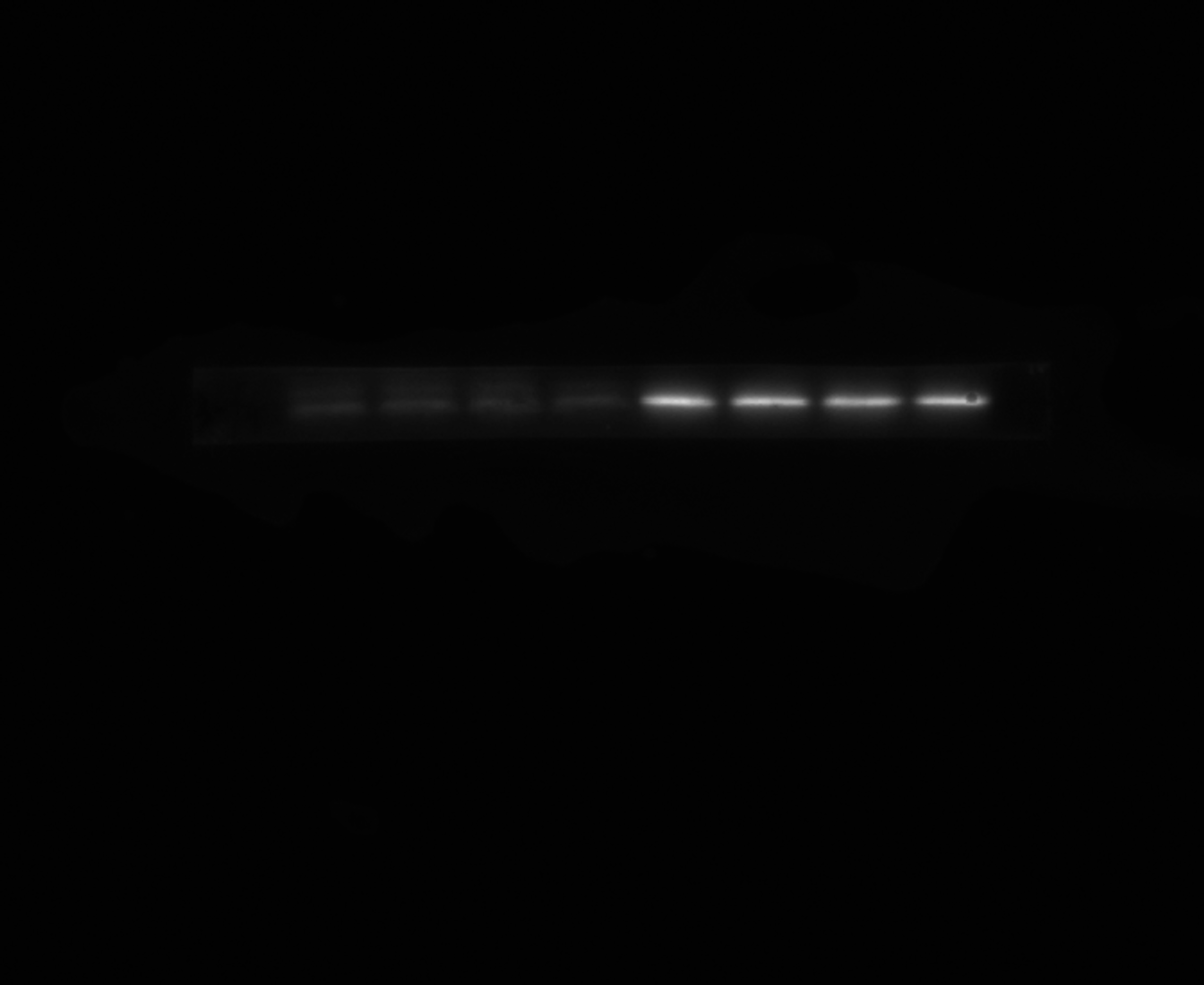


Fig 1D-β-actin


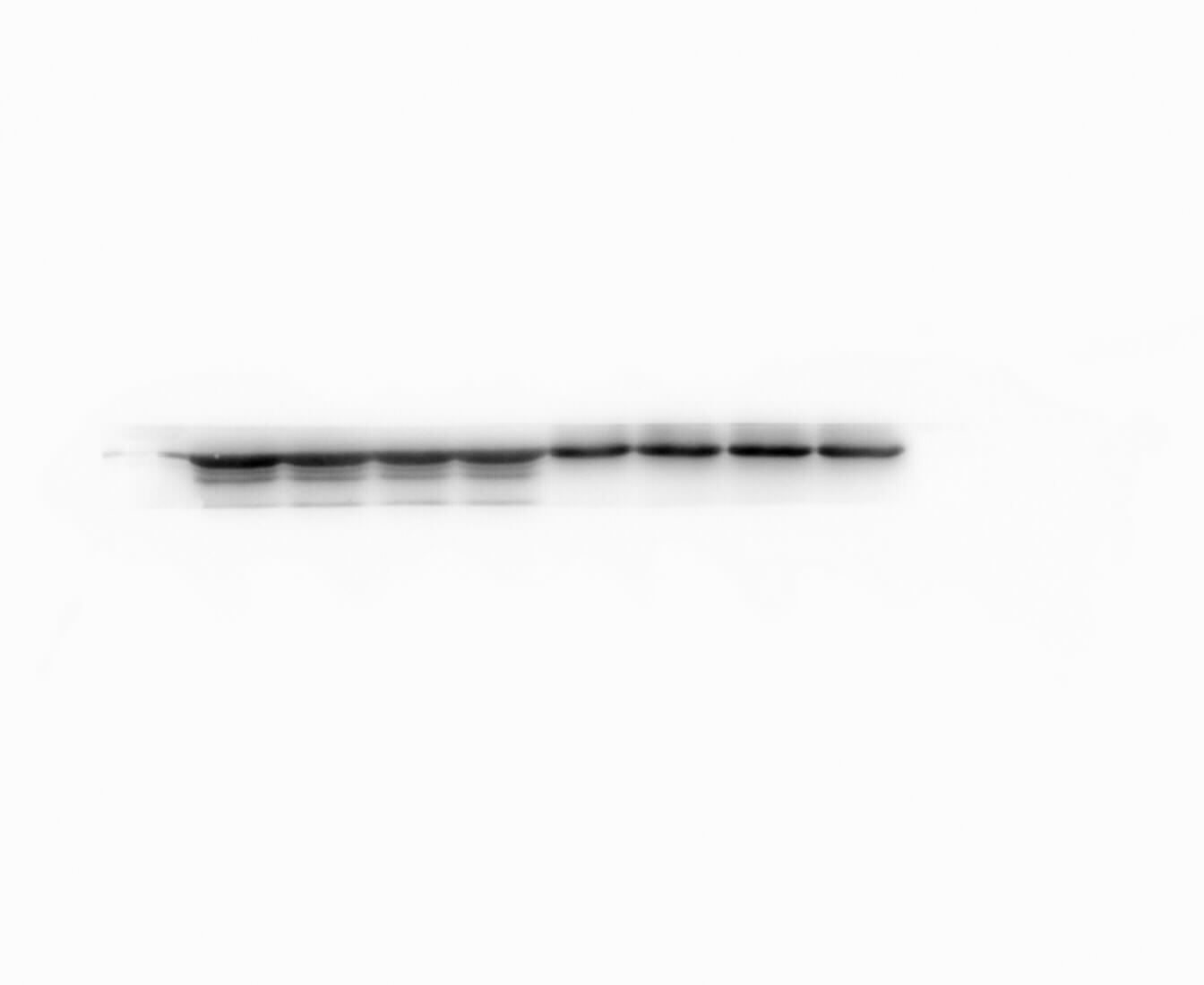

Supplement: Supplementary file 1 [file Supplementaryfile1.docx]
