# Supplementary material for: Adipocyte fatty acid-binding protein 4 suppresses contraction of mouse ventricular myocytes via a calcium-independent pathway
Source: Front Physiol. 2026 Mar 10;17:1682010. doi: 10.3389/fphys.2026.1682010 (PMC13008700; doi:10.3389/fphys.2026.1682010)
Supplement: Supplementary file 2 [file Table1.docx]

| Gene | Forward primer sequence (5′-3′) | Reverse primer sequence (5′-3′) |
| --- | --- | --- |
| FABP3 | AGTCACTGGTGACGCTGGACG | AGGCAGCATGGTGCTGAGCTG |
| FABP4 | TTGGTCACCATCCGGTCAGA | CCTGTCGTCTGCGGTGATTT |
| 18SrRNA | CGCCGCTAGAGGTGAAATTC | CCAGTCGGCATCGTTT ATGG |

The thermal cycling program was as follows: denaturing at 95*°*C for 10 s, followed by 40 cycles of 95 °C for 5 s, annealing at 65*°*C for 30 s, and extension at 72*°*C for

20s.
